# A Rare Case of Sarcoidosis With Urologic Symptoms As the Presenting Feature

**DOI:** 10.7759/cureus.28315

**Published:** 2022-08-23

**Authors:** Ana Luísa Campos, Magda Costa, Filipa Gonçalves, Clarisse Neves, Jorge Cotter

**Affiliations:** 1 Internal Medicine, Hospital da Senhora da Oliveira, Guimarães, PRT

**Keywords:** fertility, testicular cancer, non-caseating granuloma, genitourinary tract, sarcoidosis

## Abstract

Sarcoidosis is a multisystem idiopathic disease that can affect virtually any organ of the human body. However, genitourinary tract involvement is rare. We describe the case of a 33-year-old man with post-coital right scrotal pain. Scrotal ultrasound showed two vascularized nodular lesions in the right testicle and one in the left. A thoracic and abdominopelvic computed tomography scan showed micronodular infiltrate in the liver, spleen, lungs, and millimetric retroperitoneal and iliac lymph nodes. Levels of alpha-fetoprotein and human chorionic gonadotropin beta were normal. On positron emission tomography, the previously documented micronodular infiltrate exhibited features suggestive of an inflammatory etiology. The aspiration cytology of an iliac ganglion was described as normal, with no malignant cells. A liver biopsy revealed non-caseating epithelioid granulomas characteristic of granulomatous hepatitis. After exclusion of other causes of granulomatous inflammation, conjugation of clinical and histological features led us to the diagnosis of sarcoidosis with pulmonary, hepatic, splenic, and genitourinary involvement. This clinical report describes one of the rare occasions when the reproductive tract is affected by sarcoidosis and is the first organ to present signs of involvement by the disease, which reinforces the importance of considering sarcoidosis in the differential diagnosis of urologic conditions. The diagnosis of testicular sarcoidosis is challenging and the issue of its impact on fertility is particularly important.

## Introduction

Sarcoidosis is an idiopathic inflammatory systemic disease thought to result from genetic predisposition and environmental factors, whose main feature is the presence of non-caseating granulomas: compact clusters of epithelioid cells and multinucleated giant cells with little or no central necrosis often surrounded by lymphocytes [1]. This disorder affects one to six in every 1000 people around the world. It is three to 20 times more common in African Americans than in Caucasians, and approximately 10 times more common in women than in men. Most patients are asymptomatic. Generally, the suspicion of sarcoidosis arises when bilateral hilar adenopathies (a feature present in 90% of patients with sarcoidosis) are incidentally found on chest radiograph [2].

The diagnosis of sarcoidosis is confirmed when all the following criteria are fulfilled: compatible clinical and radiological presentation; the presence of non-caseating granulomas in the histopathological exam; exclusion of other causes of granulomatous inflammation [1,3]. As sarcoidosis is a diagnosis of exclusion, the sampled tissue must be carefully examined to exclude other causes of granulomatous inflammation, such as fungal and mycobacterial infections, and foreign body reactions [1,2].

Although lung and intrathoracic lymph nodes are the most affected organs, sarcoidosis can virtually affect any organ in the body. The most commonly involved extrathoracic organ is the skin [1]. On the other hand, the involvement of the genitourinary tract is considered rare [4]. It occurs in less than 0.2% of all clinically diagnosed cases of sarcoidosis, with the epididymis, followed by testicles, being the most frequently involved sites [2].

Clinical presentation is related to the involved organs. Patients with testicular involvement usually present with a non-painful, diffuse nodular mass [2]. Testicular cancer and sarcoidosis have their peak incidence in the same age group and a similar presentation, so they can be very difficult to distinguish [4,5]. Therefore, although rare, genitourinary sarcoidosis should be part of the differential diagnosis of urological conditions [5]. The purpose of this article is to describe an atypical and rare presentation of sarcoidosis that can mimic malignant urological conditions and lead to delay in diagnosis and unnecessary diagnostic and therapeutic procedures, with important consequences, especially regarding to future fertility.

## Case presentation

Before the internal medicine consultation

A 33-year-old man was seen in the internal medicine outpatient consultation to study testicular nodularities. Two years before, he started complaints of post-coital right scrotal pain. A scrotal ultrasound showed irregular contours and heterogeneous echostructure of the right epididymis. Symptoms resolved with a prescription of non-steroidal anti-inflammatory drugs for an epididymitis diagnosis. One year later symptoms recurred. A new scrotal ultrasound was performed, revealing three vascularized testicular lesions, two in the right testicle and one in the left.

The patient was admitted to the urology inpatient department to continue the study. Abdominopelvic computed tomography (CT) showed a hypodense micronodular hepatic infiltrate, as well as several infracentimetric hypodense micronodular images in the spleen, and retroperitoneal and right external iliac millimetric lymph nodes, with characteristics not suggestive of metastatic disease. The thoracic CT showed bilateral parenchymal micronodules predominantly on the right, totally nonspecific. The levels of α-fetoprotein (α-FP) and β-human chorionic gonadotropin (β-HCG) were normal. Blood analysis revealed a slight elevation of liver enzymes as shown in Table 1.

**Table 1:** Blood analysis results

Test result		Reference values
Hemoglobin	16.0	14.0 – 18.0 (g/dL)
Mean corpuscular volume	81.9	83 – 103 (fL)
Mean corpuscular hemoglobin	29.3	28 – 34 (pg)
Mean corpuscular hemoglobin concentration	35.8	32.0 – 36.0 (g/dL)
White blood cells	10	4.8 – 10.8 (x 10^3^/µL)
Neutrophils	6.2	1.8 – 7.7 (x 10^3^/µL)
Eosinophils	0.2	0.00 – 0.49 (x 10^3^/µL)
Basophils	0.1	0.0 – 0.1 (x 10^3^/µL)
Lymphocytes	3.5	1.0 – 4.8 (x 10^3^/µL)
Monocytes	1.0	0.12 – 0.80 (x 10^3^/µL)
Platelets	333	150 – 350 (x 10^3^/µL)
C-reactive protein	3.6	<3.0 (mg/L)
Urea	43	15 – 39 (mg/dL)
Creatinine	0.90	0.70 – 1.30 (mg/dL)
Sodium	139	135 – 146 (mEq/L)
Potassium	4.40	3.5 – 5.1 (mEq/L)
Total bilirubin	0.91	0.3 – 1.2 (mg/dL)
Direct bilirubin	0.16	0.0 – 0.3 (mg/dL)
Aspartate aminotransferase	55	12 – 40 (UI/L)
Alanine aminotransferase	119	7 – 40 (UI/L)
Gamma-glutamyl transferase	158	0 – 73 (UI/L)
Alkaline phosphatase	153	46 – 116 (UI/L)
Lactate dehydrogenase	368	120 – 246 (UI/L)

**Figure 1:**
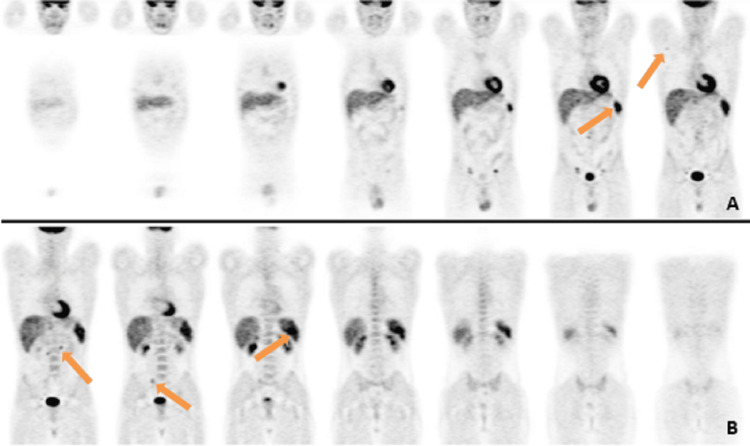
Images of the entire body fluorodeoxyglucose positron emission tomography A: Showing from left to right, fluorodeoxyglucose uptake (orange arrows) by the spleen and an axillary ganglion; B: From left to right, fluorodeoxyglucose uptake (orange arrows) by infradiphragmatic adenopathies and the spleen

Serological tests for hepatitis B virus (HBV), hepatitis C virus (HCV), and human immunodeficiency virus (HIV) were negative. The levels of angiotensin-converting enzyme (ACE), immunoglobulins, and sedimentation rate were normal. The patient underwent an entire body fluorodeoxyglucose positron emission tomography (FDG-PET) which showed mild to moderate increased FDG anomalous uptake by infra-diaphragmatic adenopathies, in a small right axillary ganglion and the spleen, suggestive of an inflammatory etiology (Figure 1).

Aspiration of an inguinal ganglion was performed. Cytological analysis revealed it to be a heterogeneous lymphoid cell population with the presence of small and intermediate-sized and occasionally aggregated lymphocytes, outlining lymphoid follicle morphology, consistent with an origin in lymph node parenchyma. Cellular elements of an epithelial nature were not identified, excluding a metastatic neoplastic process.

Given the duration of symptoms, the bilateral testicular involvement, the normal levels of α-FP and β-HCG and the results of the other diagnostic tests performed until then, the probability of primary testicular neoplasm was low. An internal medicine outpatient consultation was requested for further etiological investigation.

Management in the internal medicine consultation

At the time of the first internal medicine appointment, the patient no longer had post-coital scrotal pain, but he continued to feel slight pain on scrotal palpation. He denied changes in urine characteristics, fever, hyperhidrosis, anorexia, weight loss, fatigue, myalgias, chills, changes in the skin, mucous membranes, eyes, and pain or inflammatory signs of the joints. He reported a long-standing dry cough but had no dyspnea or chest pain. There was no history of contact with domestic animals or livestock, consumption of unpasteurized dairy products, or non-piped water. He had not traveled to the mountain or the countryside. He was a nonsmoker and reported no risky sexual behavior. He was not aware of any close contact with known cases of tuberculosis.

**Figure 2:**
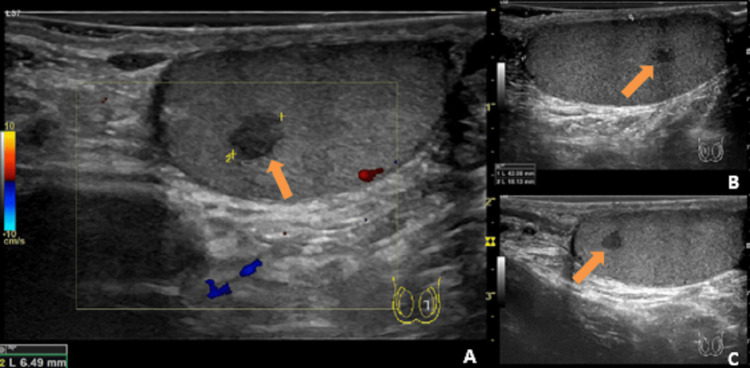
Images of the scrotal ultrasound showing three nodular lesions (orange arrows) A: One nodular lesion on the left testicle, B & C: Two nodular lesions on the right testicle

The patient had no scrotal inflammatory signs. Some irregularity was noticeable on palpation of both testicles, especially in the right. No other abnormalities were found on the physical examination. A new ultrasound reported the three known testicular lesions with slightly smaller dimensions (Figure 2).

The syphilis screening test, the serological tests for Epstein-Barr virus (EBV), herpes simplex virus (HSV) 1 and 2, and for *Brucella* and *Borrelia* were negative. The patient had HBV immunity status after vaccination. He also had immunity to toxoplasmosis and rubella. A second serological test for HCV and HIV was performed, with a negative result.

The interferon-gamma release assays (IGRA) test for tuberculosis was negative. The urine chemical and microscopic analysis showed no alterations. No acid-alcohol-fast-bacilli (AAFB) were observed in the patient urine with the Ziehl-Neelsen stain. *Mycobacterium tuberculosis* deoxyribonucleic acid (DNA) screening by polymerase chain reaction (PCR), as well as urine culture tests for mycobacteria, was negative.

Blood and urine calcium levels were normal and serum protein electrophoresis showed no significant changes. Gamma-glutamyl transferase (GGT) and alkaline phosphatase (AP) remained slightly increased, while transaminases had dropped to normal values. Serum albumin, as well as prothrombin time (PT), was normal. At this time, ACE was elevated (180 U/L for a normal range of 35-90 U/L). Immunoglobulin G1 was slightly increased, while serum free light chain assay was normal.

**Figure 3:**
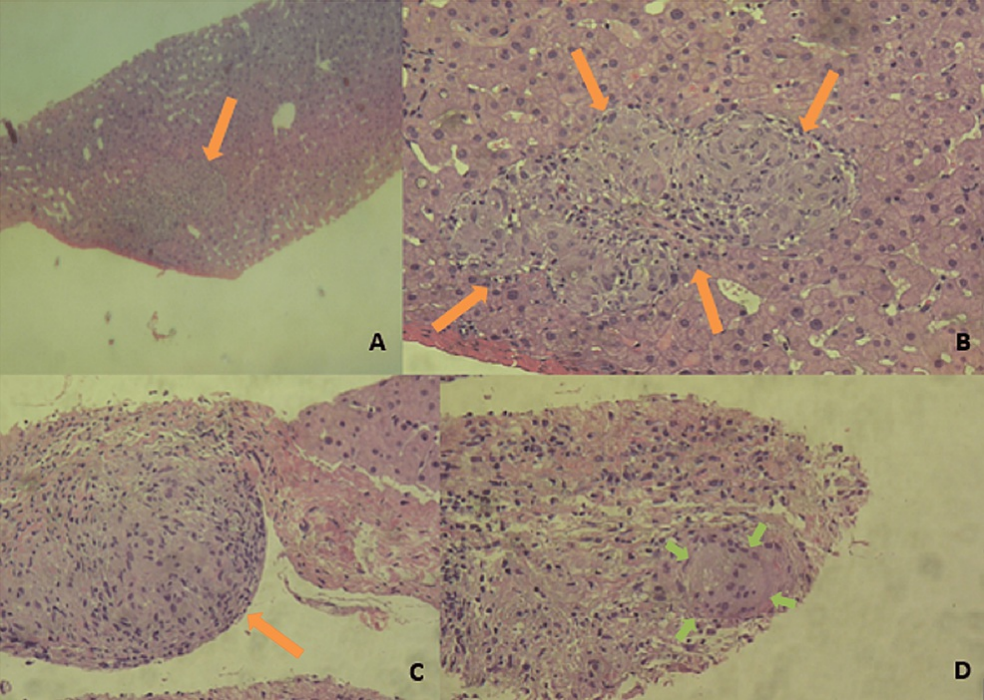
Anatomopathological analysis of the hepatic tissue showing the presence of non-caseating epithelioid granulomas (orange arrows in panels A, B, and C) and a multinucleated giant cell (green arrows, panel D).

A new thoracic and abdominopelvic CT was performed. Scattered micronodules in the hepatic and splenic parenchyma persisted, but the pulmonary nodules identified on the CT about one year before had disappeared. A liver biopsy was performed. Anatomopathological analysis showed granulomatous hepatitis, characterized by the presence of epithelioid granulomas with multinucleated giant cells (Figure 3). Ziehl-Neelsen staining was negative and no necrosis was seen.

No changes were found on fiber optic bronchoscopy (FOB). Ziehl-Neelsen staining, mycobacterial culture, and *Mycobacterium tuberculosis* DNA screening by PCR in bronchial aspirate and bronchoalveolar lavage (BAL) samples were also negative. Cytological analysis of BAL and bronchial aspirate did not reveal neoplastic cells. The diagnosis of sarcoidosis was established.

To assess the involvement of other organs by sarcoidosis, further investigation was carried out. The electrocardiogram, echocardiogram, cranioencephalic CT scan, and spirometry showed no structural or functional alterations. The ophthalmologic evaluation did not show any pathological findings. Thyroid stimulating hormone, thyroxine, parathyroid hormone, and vitamin D levels were within the normal range.

The patient has no children. The potential implications of the disease on fertility were explained to the patient, who understood the need to start with a spermogram to understand their fertility status. As he does not wish to have children, he chose not to proceed with the investigation on his fertility status and he does not want specific assistance regarding fertility.

As the patient was asymptomatic and without signs of disease activity, no therapy was introduced. A regular follow-up was maintained. After two years of follow-up on the internal medicine outpatient consultation, the patient did not report new symptoms, remains without genitourinary symptoms, and the dry cough resolved. The most recent scrotal ultrasound continues to show bilateral testicular lesions with stable dimensions. The abdominopelvic and renal ultrasound shows a slightly heterogeneous hepatic parenchyma without focal lesions; a spleen of normal dimensions with a homogeneous structure and without focal lesions; and no retroperitoneal adenopathies or changes in the kidneys, prostate, or other organs were found. The most recent chest X-ray shows no changes, and the ACE is within normal range. Liver enzymes normalized, and there is no increase in blood or urine calcium levels.

## Discussion

The involvement of the reproductive tract by sarcoidosis is rare, affecting less than 0.2% of men in life and being found in 5% of men at autopsies [4]. By 2004, 60 cases of sarcoidosis involving the male reproductive tract had been reported [6]. And over 14 years, between 2004 and 2018, we found only eight more cases described in the literature [7]. In this article, we describe a rare case in which testicular sarcoidosis was the presenting manifestation of the systemic disease [4].

Testicular sarcoidosis typically affects African Americans between the ages of 20 and 40. Usually, when the testicles are affected, there is also sarcoid disease in the epididymis, but there are cases of isolated testicular disease. Testicular sarcoidosis usually presents as a painless nodular mass in one of the testicles [2]. In the present case, we found masses in both testicles, which is also common [4]. Scrotal masses due to sarcoidosis can mimic infection or malignancy. The major concern in testicular sarcoidosis is that the peak incidence of the disease between 25 and 35 years of age, is also the peak incidence of malignant tumors of the testicles [7]. Obviously, other differential diagnoses such as tuberculosis, syphilis and lymphoma also have to be excluded [8].

Testicular cancer traditionally occurs at the same age group of sarcoidosis, but is more common in Caucasians [2]. Distinguishing genitourinary sarcoidosis from testicular cancer can be very difficult, but it is absolutely critical, as a wrong diagnosis can, on one hand, lead to unnecessary surgical interventions with serious implications for fertility; or, on the other hand, cause a delay in the treatment of malignant conditions [5]. Epidemiological data, imaging tests, clinical presentation, serum markers (ACE, α-FP, β-HCG, lactate dehydrogenase) and histological analysis of the affected tissue help to clarify the diagnosis [5, 7]. In the case of testicular sarcoidosis, the histological analysis implies surgical procedures with potential impact on fertility, which constitutes a limitation to the diagnosis.

Hypercalciuria (which is more common than hypercalcemia) and elevated ACE levels are suggestive of sarcoidosis [2]. The ACE is reported to be elevated in 75% of untreated subjects with active sarcoidosis, whereas levels are normal in patients with testicular cancer. In about 50% of patients with non-seminoma-type testicular malignancies, β-HCG and α-FP are elevated, which is not the case in sarcoidosis [5]. However, serum markers have a limited role in diagnosis. Elevated ACE levels, which were thought to be sarcoidosis-specific and related to disease activity, actually have low sensitivity and specificity and achieve false-positive rates of about 15% [1]. Imaging tests do not distinguish sarcoidosis from malignancy [7]. On ultrasound, sarcoid testicular masses usually appear as hypoechoic lesions, but this feature is not specific of sarcoidosis [2,7]. Positron emission tomography scanning has gained an important value in the diagnosis and management of sarcoidosis, since it allows the detection of silent disease, helping to define the best site for biopsy [3].

The diagnosis of sarcoidosis is based on three fundamental assumptions: the presence of non-caseating granulomas in the histopathological analysis (without organisms or particles); a compatible clinical presentation; and exclusion of other causes of granulomatous inflammation [1,3]. There are no pathognomonic diagnostic tests for sarcoidosis, so the diagnosis remains as one of exclusion [3]. As the differential diagnosis of granulomatous inflammation is extensive, the definitive diagnosis of sarcoidosis in our patient was preceded by a comprehensive and lengthy diagnostic process.

Histopathological confirmation is necessary to establish the definitive diagnosis of sarcoidosis. In general, if the signs and symptoms are compatible, the presence of non-caseating granulomas in at least one organ is sufficient for the diagnosis, and the involvement of the other organs is assumed. This was particularly important in the case of our patient, as we chose to biopsy an organ that was more accessible and had less potential for complications, especially with regard to future fertility. Liver biopsies have over 90% sensitivity in detecting granulomas [1]. In the case of this patient, the signs and symptoms, all the previous investigation and the result of the liver biopsy allowed a definitive diagnosis: sarcoidosis with testicular involvement.

Spontaneous remission of sarcoidosis occurs in many cases over a period of three years without any permanent damage to the tissues involved, so most patients do not need treatment [6]. Given the frequent occurrence of spontaneous remission and the potential adverse effects associated with corticosteroids, which remain the first line therapy, treatment for sarcoidosis is generally only initiated if vital organ function is threatened or if hypercalcemia is present [1,3,6]. Some aspects in the case of this patient call our attention to spontaneous remission, such as the resolution of symptoms, the size reduction of testicular lesions and the complete return of liver enzymes to normal values.

Although the reproductive organs are not vital organs, there is a concern about fertility, which interferes with the decision to treat. Fibrosis and occlusion associated with granulomatous inflammation in the epididymis can lead to oligospermia or azoospermia. In these cases, corticosteroid therapy can lead to a reduction in the size of the granulomas and, consequently, their obstructive effect on the epididymis, improving sperm counts [9]. This was not the case for our patient, but for patients who wish to have children, fertility should be investigated through a spermogram, as corticosteroid therapy can significantly improve sperm quality [2,8].

Another concern in sarcoidosis follow-up is its association with cancer. Sarcoidosis may be associated with an increased incidence of several types of solid tumor malignancies, including testicular cancer, and lymphoproliferative disease [2,4,5]. However, it has been suggested that the sarcoid reaction may be part of the body's response to the tumor and not the event responsible for the onset of the neoplasm. We can find tumor-related sarcoid reactions in 4% of sarcomas and at higher rates in lymphomas [2]. This association gives additional strength to the idea that patients with sarcoidosis should be followed up regularly and on a long-term basis. After three years of follow-up, our patient still shows no clinical, analytical or imaging signs of active sarcoidosis or its association with malignancy.

## Conclusions

The diagnosis of sarcoidosis continues to be a challenge for physicians since the disease can present itself in a very different form and be confused with multiple other diseases. The presence of non-caseating granulomas is the basis of the diagnosis, but, as it is not pathognomonic, requires an exhaustive diagnostic process.

Although genitourinary sarcoidosis is rare and even rarer in the presentation of the systemic disease, it should always be considered in the differential diagnosis of urological conditions to avoid unnecessary procedures like orchiectomy that has an important impact on future fertility. Generally, the treatment of sarcoidosis is only indicated when there is dysfunction of vital organs, but when we are talking about the involvement of the reproductive organs, the issue of fertility is a concern that also influences the decision to treat.
